# The Spatial Effect of Air Pollution Governance on Labor Productivity: Evidence from 262 Chinese Cities

**DOI:** 10.3390/ijerph192013694

**Published:** 2022-10-21

**Authors:** Fei Ren, Yuke Zhu, Dong Le

**Affiliations:** School of Economics, Zhongnan University of Economics and Law, Wuhan 430073, China

**Keywords:** air pollution governance, labor productivity, spatial effect, alleviate endogenous problems, urban innovation capacity, residents’ health, joint prevention, governance mechanism

## Abstract

According to epidemiological studies, air pollution can increase the rate of medical visits and morbidity. Empirical studies have also shown that air pollutants are toxic to animals. Using data from 262 Chinese cities for the period 2005 to 2018, this study systematically investigated the spatial spillover effect and transmission mechanism of air pollution governance on urban labor productivity. In this study, we also explored the changing trend of labor productivity in China from a dynamic perspective. Additionally, we selected the air flow coefficient and environmental regulations as two instrumental variables of air pollution governance to effectively alleviate endogenous problems existing in the model. The results show that air pollution governance plays a significant role in promoting the improvement of labor productivity. The effect of air pollution governance on labor productivity in eastern cities is better than that in central and western cities, and its effect in developed cities is better than that in undeveloped cities. With the increased intensity of air pollution governance, its effect on labor productivity is also strengthened. Urban innovation capacity and residents’ health are important channels for air pollution governance in the promotion of labor productivity. Finally, this study proposes policy recommendations, such as implementing a joint prevention and governance mechanism, as well as improving air pollution prevention and government regulations.

## 1. Introduction

According to the report of China’s ecological and environmental conditions in 2020, only 201 cities (59.9% of the total number of cities) in the country had achieved the specified air quality standards. Additionally, air pollution causes 1.2–1.4 million deaths every year [1] and leads to direct and indirect economic losses that account for 1–8% of China’s gross domestic product (GDP). Serious air pollution conditions cause huge economic losses and harm people’s health. Such conditions not only hinder urban economic development, the damage to residents’ health is also irreversible [2,3]. In 2018, the government pledged to resolutely curb the damage to the air environment. However, local governments often neglect the prevention and governance of air pollution when developing the regional economy. In western China, excessive pollution emissions and low energy utilization efficiency are obvious. In addition, governments in adjacent cities “pass the buck” to each other for air pollution governance. Thus, the central government’s air pollution governance measures are not accurately implemented. So how to improve urban air quality is an urgent problem to be solved.

As an important subject of pollution governance, enterprises’ production and operation will be affected by environmental regulations. The raising of environmental regulations will force enterprises’ to improve technological innovation ability [4]. On the one hand, environmental regulation can reduce the pollution discharge of enterprises and correct the management process to improve labor productivity [5]. On the other hand, the introduction of cleaner production technology will lead to an increase in production costs, which will reduce the enterprises’ labor productivity [6]. In addition, there may be more complex nonlinear relationships between environmental regulation and labor productivity [7].

As one of the environmental regulation measures, whether air pollution governance can improve labor productivity and achieve sustainable development is an urgent issue. In order to solve the above problems, this study selected the Spatial Dubin Model to investigate the spatial correlation between air pollution governance and labor productivity, conducted a series of robustness tests on the research results, and excluded the possible endogeneity problems. In the mechanism analysis, the path of air pollution governance affecting labor productivity is further investigated. The results show that air pollution governance affects labor productivity mainly through two paths: urban innovation ability and residents’ health. The results of heterogeneity analysis show that the implementation of air pollution governance policies has certain differences. Therefore, the correlation between air pollution governance and labor productivity is of great theoretical value and practical significance for China’s economic sustainable development.

### 1.1. Literature Review

#### 1.1.1. Visual Analysis of the Literature

Based on the literature analysis, which included works from the Web of Science database, an in-depth analysis of the literature characteristics and trends in air pollution governance was conducted in this study. The number of published documents focusing on quantitative statistics and the “hot” trends in air pollution governance were analyzed through co-words, cluster, and burst detection analyses. The main principle of a co-word analysis is the number of times that keywords appear together in the same document. A cluster analysis classifies keywords according to their relationship strength, gathers those with a close relationship and strong correlation to form a cluster, and then explores the research hot spots of the disciplines they represent [8]. A research frontier is usually represented as a group of emergent dynamic concepts and potential research questions. A burst detection analysis can be used to understand the characteristics of a temporal distribution and dynamic variation of emergent words, which can better reflect the research frontier and development trend in the knowledge domain.

#### 1.1.2. Overall Growth Trend Analysis

**Figure 1:**
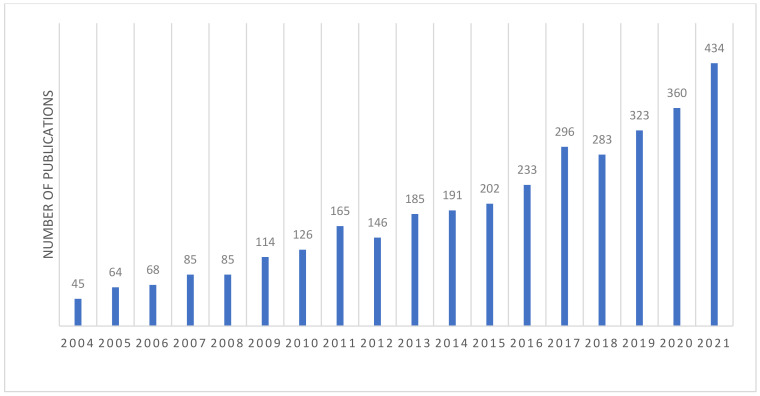
Number of articles regarding air pollution governance published in past years.

Figure 1 shows the number of articles regarding air pollution governance published from 2004 to 2021 based on the Web of Science core study database. The number of papers on air pollution governance has been increasing annually, and greater attention to this subject in academic circles has been noted.

#### 1.1.3. Analysis of Highly Cited Literature

**Figure 2:**
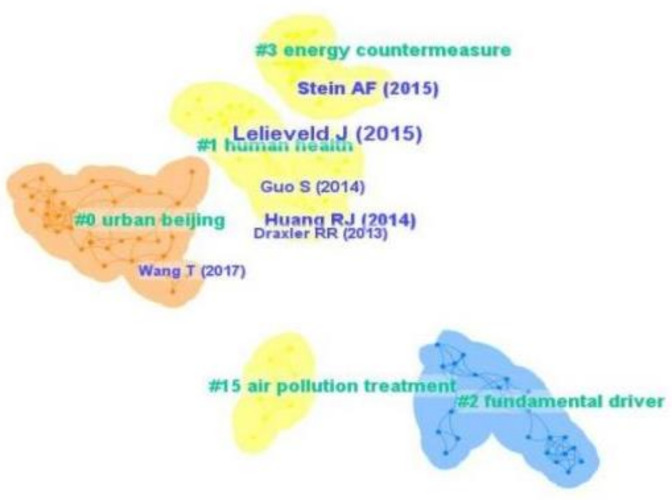
Co-citation map of air pollution governance for the period 2000 to 2021.

The co-citation map of air pollution governance was drawn with CiteSpace software (Figure 2). By reviewing cited literature about air pollution governance, the foundational literature and theoretical basis in this field were analyzed. In terms of high citation frequency, 6 of the 411 studies were cited 5 or more times. From the perspective of themes, relevant situational factors, such as urban Beijing, air pollution treatment, human health, energy countermeasures, and the fundamental driver, have received the most attention in this field. Lelieveld (2005) was cited 12 times, followed by Huang (2014) (8 times), and Stein (2015) (7 times).

#### 1.1.4. Keyword/Co-Word Visual Analysis

**Figure 3:**
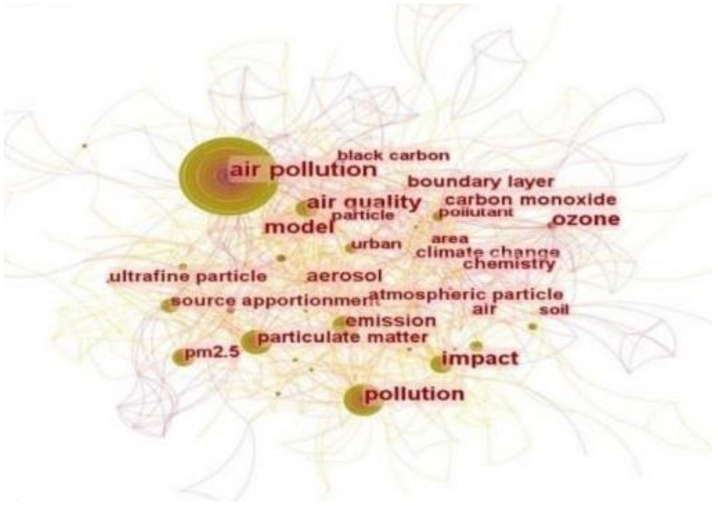
Co-occurrence map of air pollution governance for the period 2000 to 2021.

The visualization function of CiteSpace and interpretation of the keyword co-occurrence maps can facilitate an in-depth analysis of keywords from a large number of articles and promote a deeper understanding of the important themes. The co-occurrence maps of air pollution governance for the period 2000 to 2021 is shown in Figure 3. The node size is positively correlated with the frequency of keyword occurrence—that is, the larger the node, the higher the frequency of keyword occurrence. In Figure 3, we show that air *pollution, pollution, air quality, carbon monoxide impact, climate change, black carbon, source, particulate matter*, and *ozone* were the most frequently used keywords in the studies of air pollution governance from 2000 to 2021.

#### 1.1.5. Burst Detection

**Figure 4:**
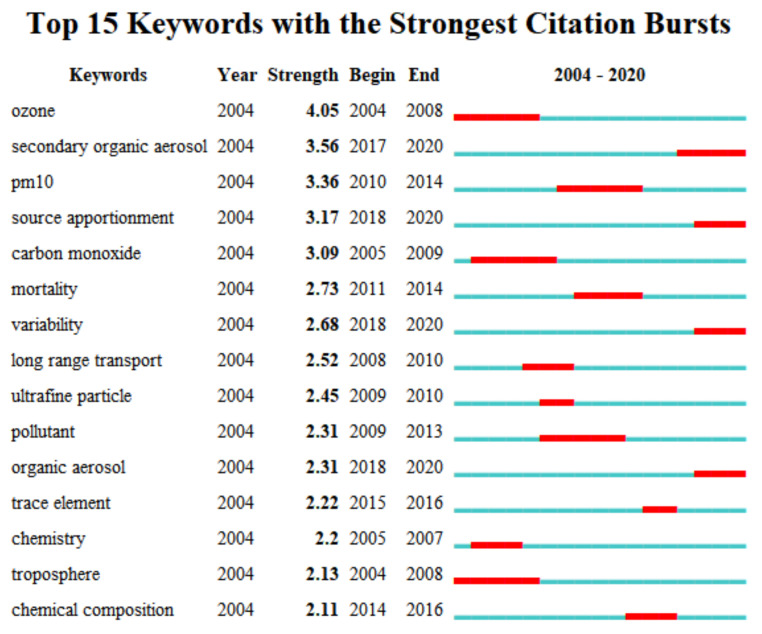
Keywords in the field of air pollution governance.

Burst detection is typically used as a computational technique to identify abrupt events or significant information. Figure 4 shows the top 15 keywords with the strongest citation bursts. The blue lines represent time intervals, and the red segmented lines denote bursts and indicate the beginning and ending years.

From the analysis of keywords in Figure 4, the field of air pollution governance is diversified. Different words have emerged in different periods, among which the strongest keyword is *ozone*, with the strongest score as high as 4.05. The longest burst detection time, a duration of four years, was associated with ozone, PM10, carbon monoxide, haze, and troposphere,. It is worth mentioning that the following keywords have emerged in the past six years: chemical composition, trace element, secondary organic aerosol, source apportionment, variability, and organic. The indication is that air pollution governance is a research topic that has been actively analyzed recently, and it may become a research focus and hot topic in the future. In terms of practical applications, chemical composition, trace elements, secondary organic aerosol, source apportionment, and organic aerosol are mainly used to study the components of air pollution. According to the variations in air pollution components and governance methods, governance measures should be adjusted in a timely manner.

### 1.2. Air Pollution Governance and Labor Productivity

The negative impact of air pollution has also been emphasized in recent years. Air pollution not only has irreversible consequences for residents’ health, but it also seriously hinders regional economic development [9]. Existing studies mainly discuss the reduction in labor productivity caused by air pollution from a microscopic perspective. When air pollution is eliminated, labor productivity will significantly improve. Unfortunately, existing studies are mainly focused on a certain enterprise or specific industry, such as the pear packaging factory workers [10], athletes [11], textile workers [12], Ctrip employees [13], and prisoners [14]. The micro individual’s labor productivity is easy to measure. However, owing to the narrow sample selection range, air pollution governance cannot reflect real relationships between air pollution governance and labor productivity. However, some studies explored the effect of air pollution governance on labor productivity from specific environmental regulation policies. Wang et al. [15] found that in the short and long term, the *Ambient Air Quality Standards (2012)* significantly reduced the concentration of PM2.5 and SO2 in cities and improved the health of urban residents. Gu and Yan [16], through specific air pollution governance measures in the *Ambient Air Quality Standards (2012)* found that air pollution governance can significantly improve enterprise productivity.

Additionally, there are still some studies from a macro perspective that explored the impact of air pollution governance on labor productivity. Zhou and Li [17] found that air pollution significantly worsens income distribution in China and they also proved that increasing health expenditures and declining labor productivity mediate the effect of air pollution on income distribution. Through research, He and Ji [18] found that the cause of labor productivity loss was the impact on the health of employees. Moreover, when employees were exposed to more PM2.5, the physical activity index decreased significantly and the disease prevalence increased. Farzanegan et al. [19] found that air pollution has a positive and significant effect on outmigration. They also propose air pollution harmfully impacts the physical and mental health of citizens, reducing labor productivity and student academic performance. However, the existing research neglects spillover effects, and their conclusions cannot reflect the real economic laws.

### 1.3. Summary

Air pollution has seriously affected urban residents’ health and economic development. Is there a significant spatial spillover effect of air pollution governance? What is the mechanism by which air pollution governance promotes labor productivity? Deficiencies remain in the existing research on these issues. First, the existing literature mostly ignores the spatial spillover effect of air pollution governance in terms of related issues. That is, how will the governance of air pollution in nearby areas affect local areas’ labor productivity? Second, the existing studies of air pollution governance on labor productivity have been conducted primarily from a static perspective, but not from the long-term and short-term dynamic perspectives. Third, the existing literature mostly ignores the endogeneity problem in the model when studying air pollution, which makes the research conclusions deviate from the actual situation. Based on the above shortcomings, we used the main emissions data to systematically investigate the spatial spillover effect of air pollution governance on labor productivity and its transmission mechanism from the perspectives of region, time, and urban development level. While replacing the explanatory variables and weight matrix for the robustness test, the DSDM was used to study their robustness from long-term and short-term perspectives. Two instrumental variables, air flow coefficient and environmental regulations, were selected to alleviate the endogeneity problem of the model when analyzing the effects of air pollution governance.

## 2. Theory

Air pollution governance mainly affects labor productivity through the spatial spillover effect, urban innovation ability, and residents’ health levels. Therefore, in this study, we analyzed the mechanisms underlying these factors.

### 2.1. Air Pollution Governance Affects the Labor Productivity of Adjacent Cities through the Spatial Spillover Effect

**Figure 5:**
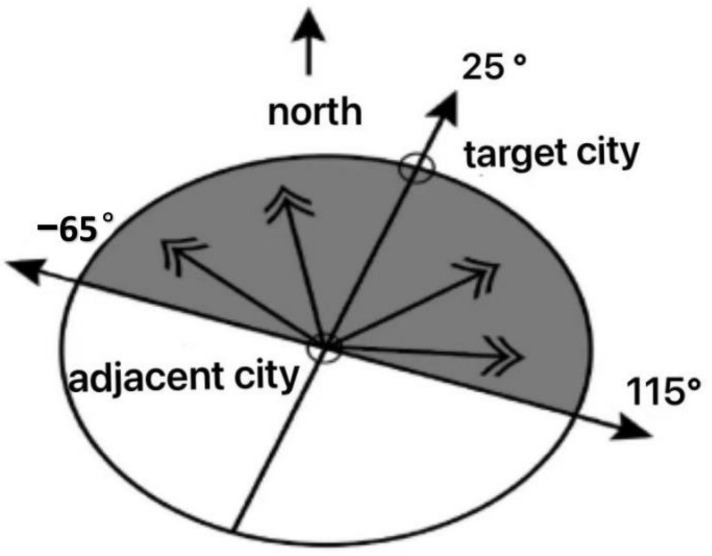
Spillover effects of air pollution based on wind direction. It means that the adjacent city’s air pollutants can significantly influence the target city.

To estimate the spatial spillover effect of air pollution, a Pollutant Decline Model is constructed to simulate it. Several concentric circles are constructed with the target city as the center. Denoted as b, b = 1, 2, 3…, the distance between each concentric circle is 50 km, the distance between the circle and the center of the circle is 0 to 50 km, then b is 1, 50 to 100 km, b is 2, the rest can be conducted in the same manner. If the circle is 1200 km from the center, and b is 36, then the amount of air pollutants from adjacent areas to the target city is as follows:

(1) $$\begin{array}{l} P_{td}^{f}=I_{b}[\lambda_{1b}+\lambda_{2b}abs[\cos\theta_{td}^{fn}]p_{td}^{n}]+\lambda_{3}w_{td}^{f}+\omega_{f}+k_{rtm}+\varepsilon_{td}^{fn},\\ \forall f,n\in F,n\;\neq f,\forall b=1,\ldots 36 \end{array}$$

where F is the urban collection, $p_{td}^{f}$ is the air pollution concentration of target city F, and $p_{td}^{n}$ is the air pollution concentration of the surrounding city N. $w_{td}^{f}$ represents the impact of weather on air pollution spillovers. When the surrounding cities are in concentric circles b, $I_{b}$ is denoted as 1; otherwise, as 0, $\lambda_{1b}$ is the intercept term of each distance segment, $\lambda_{2b}$ is the coefficient of each distance segment representing the physical distance of the adjacent areas moving toward the center of the circle. $abs[\cos\theta_{td}^{fn}]$ represents a weighting of pollutants from adjacent areas to the target city. $\theta_{td}^{fn}$ represents the angle between the wind direction of the target city. $k_{rtm}$ represents the seasonal factors that affect the spillover effects of air pollutants from adjacent areas. $w_{td}^{f}$ represents the effect of precipitation and temperature. $\varepsilon_{td}^{fn}$ is error [20]. For example, if the target city is 25 degrees east of the north, assume the wind is −10 degrees west of the north, so $\theta_{td}^{fn}$ is 35 degrees west of the north. If the wind is 43 degrees north of the east, $\theta_{td}^{fn}$ is 18 degrees north of the east. So as long as the surrounding direction city with the target city’s angle is within 90 degrees, it will be exposed to air pollution from the adjacent areas. In Figure 5, the angle between the target city and adjacent cities is 25 degrees east of the north. So as long as $\theta_{td}^{fd}\in [-65{}^{\circ},115{}^{\circ}]$, it will be exposed to air pollution from the nearby areas.

Therefore, air pollution governance in adjacent areas will not only affect the labor productivity of these areas but also affect the labor productivity in local areas. On the one hand, when air pollution is governed in adjacent areas, the improvement of emission standards forces enterprises to actively develop green production technologies and introduce advanced management modes. Because advanced management experience, innovative talent, and advanced green technology have a spillover effect, green production technology is non-exclusive, which makes the advanced science and technology, talent, and management experience of neighboring regions spill over through the transregional flow of labor and machinery, thus promoting regional improvement in labor productivity. On the other hand, when air pollution governance is carried out in adjacent areas, some polluting enterprises will be forced to relocate, further strengthening the spillover effect of air pollution, which has adverse effects on residents’ health, enterprises’ production, and transportation. Finally, the spatial spillover effect of air pollution governance in adjacent areas depends on the size of the two effects, which was investigated empirically in this study.

### 2.2. Air Pollution Governance Strengthens Urban Innovation Capacity and Improves Labor Productivity

#### 2.2.1. Air Pollution Governance Forces Enterprises to Strengthen Technological Innovation and Promote Labor Productivity

In unregulated and unconstrained production conditions, increasing enterprise input can achieve output growth by using the natural state of the atmospheric environment to absorb emissions. However, enterprises will reduce the introduction of clean and green technology in order to reduce production costs. Simultaneously, while developing the economy, the government requires enterprises to reduce pollution emissions, which in turn forces them to strengthen their efforts toward green production technology innovation and enhance its capacity [21,22]. Advanced green technology can optimize the allocation of resources, reduce the undesirable output in enterprises’ production processes, and cause the capital, information, and technology to shift from traditional labor-intensive enterprises to new technology-intensive enterprises. The green technology of technology-intensive enterprises has the advantage of increasing returns and lowering spillover costs, thus driving the innovative development of upstream and downstream affiliated enterprises, improving the innovation level of urban enterprises, and promoting urban labor productivity.

#### 2.2.2. Air Pollution Governance Forces Enterprises to Improve Energy Efficiency and Promote Labor Productivity

Improved energy efficiency is an inevitable measure of air pollution governance. China is a big user of coal and oil. Based on the successful experience of developed countries in air pollution governance, improving the energy efficiency of coal, oil, and other fossil fuels is particularly important. Improvements in the utilization efficiency of coal, oil, and other fossil fuels is mainly reflected in processing, combustion, vaporization, liquefaction, desulfurization, and other aspects. At present, there is still a large gap between China and developed countries in coal chemical technology, coal conversion, desulfurization, denitrification, efficient dust removal, and other related technologies. Green technology innovation forces enterprises to optimize the industrial structure, promote improvements in the efficiency of fossil fuel energy use, reduce the emission of pollutants, and effectively improve air quality. In addition, improvements in fossil fuel utilization reduce the unexpected output and resource mismatch rate in the production process, ultimately promoting urban labor productivity.

### 2.3. Air Pollution Governance Ensures the Health of Urban Residents and Improves Labor Productivity

Residents’ health is the foundation of urban development [23]. On the one hand, the higher the level of air pollution in a city, the stronger the tendency of residents to move out. Moreover, air pollution will reduce workers’ desire to participate in the labor market, thus affecting their salary levels. Urban air pollution governance will improve the level of air quality and the living utility of workers. It will also attract a large number of migrant laborers, thus increasing the stock of the urban labor force and human capital.

On the other hand, air pollution reduces productivity by causing premature deaths of employees. It will lead to a higher incidence of diseases, increasing the economic burden on enterprises and individuals. The economic loss caused by the premature death of employees can be estimated by the value of life method [24]. The estimation formula is as follows:

(2) $$DED=\sum_{i}1.68\times \frac{CDI_{i}}{CDI_{B}}\times EM_{i}$$

where DED is the economic loss caused by the premature death of employees, $CDI_{i}$ stands for per capita disposable income in REGION i, CDIB is per capita disposable income in Beijing, and $EM_{i}$ represents the number of premature deaths due to air pollution in the city i. The loss resulting from increased morbidity of laborious diseases can be measured by the Disease Cost Method. The estimation formula is as follows:

(3) $$DEI=\sum_{ij}HE_{ij}=\sum_{ij}E_{ij}\times RP_{ij}+\frac{t_{i}}{242}\times GDP_{i}.$$

(4) $$RP_{ij}=RP_{zj}*\frac{CDI_{i}}{CDI_{Z}}.$$

(5) TEL=DED+DEI.

DEI is the financial burden of disease, j is the type of disease caused by air pollution, j represents region, and $HE_{ij}$ is the economic burden caused by category j diseases in region i. $RP_{ij}$ represents the outpatient fee, or hospitalization fee, caused by class j diseases in city i. $RP_{zj}$ represents the overall outpatient fee, or hospitalization fee, of j diseases in Province Z. $E_{ij}$ represents the number of residents with j diseases caused by air pollution in Region i. TEL stands for direct economic losses caused by air pollution [25].

Therefore, the reduction in pollution gas emissions will reduce the incidence of hospitalization rate for asthma, respiratory diseases, and heart disease among urban residents, and the mortality rate of urban residents will also be greatly reduced, [26] thereby ensuring the physical and mental health of urban residents. Consequently, increases in urban health and human capital can be expected. The increase in healthy human capital advances the development of the industrial structure, thus driving the transfer of urban production factors from low value-added processing trade industries to the high value-added green technology industry. Further, urban labor productivity is also improved.

### 2.4. Models, Variables, and Data

#### 2.4.1. Model Selection

Air quality improvement can reduce the number of air pollutants scattered into the adjacent area through air circulation. Therefore, the spatial spillover effect must be considered in the study of air quality improvement. The influence of different spatial models express different mechanisms. The SEM model assumes that the air pollution governance’s effect is mainly through the error term to affect. The SLM model assumes that the labor productivity is mainly through the space interaction effects to change other cities’ labor productivity, while the SAC and SDM model takes into account the error term and the labor productivity’s spatial spillover effects. In addition, the SDM model also considers the influence of spatial interaction, that is, the improvement of labor productivity in local areas is not only affected by the local areas’ air pollution governance, but also affected by the adjacent areas’ air pollution governance. In this paper, the LR test is implemented on the model, and the results show that the corresponding *p*-value of $\lambda^{2}$ is less than 0.1, which indicates that the spatial models SLM, SEM, and SAC cannot replace SDM. So the spatial Durbin model (SDM) was used to study the impact of air pollution governance on labor productivity. This model considers the spatial interaction effect of air pollution governance in adjacent areas when studying the effect of air pollution governance on labor productivity. The model’s formula is shown in Equation (6):

(6) $$\begin{array}{l} LP_{it}=\beta_{0}+\delta W\cdot LP_{it}+\beta_{1}\cdot \ln AQG_{it}+\beta_{2}W\cdot \ln AQG_{it}+\beta_{3}W\cdot X_{control}+\beta_{4}X_{control}+\mu_{i}+\nu_{t}+\varepsilon_{it}. \end{array}$$

where LP is enterprise productivity, lnAQG is air quality governance, $\mu_{i}$ represents the region fixed effect, $\nu_{t}$ represents the year fixed effect, $\varepsilon_{it}$ is a disturbing term, W is the spatial weight matrix, and δW⋅LP and $\beta_{2}W\cdot \ln AQG$ express the spatial influence on LP and lnAQG.

The distance between cities is not the only factor affecting the spatial spillover effect; regional economic disparities also affect the degree of air quality governance. Therefore, in this study, we used the per capita gross domestic product (GDP) to construct the weight matrix of economic distance. The economic distance matrix is constructed in Equation (7) as follows:

(7) $$W_{ij}=\{\begin{array}{l} \frac{1}{\left|\bar{Y_{i}}-\bar{Y_{j}}\right|},\;i\neq j\\ 0,\;i=j \end{array}$$

Further, $\bar{Y_{i}}=\frac{1}{T-T_{0}}\sum_{T=t_{0}}^{T}Y_{it}$, Y is the GDP, $\bar{Y}$ is the average GDP, and T is the year.

In this study, we examined the effect of air quality governance on labor productivity, and the spatial spillover effect of air quality governance in adjacent areas was obvious. Therefore, the adjacent spatial matrix was used for the model’s robustness test, and the elements of the adjacent matrix were constructed as follows: The main diagonal elements of the matrix are all 0, and the elements on the non-main diagonal are $1/d^{2}$, where d is the distance between two cities.

#### 2.4.2. Spatial Correlation Test

Moran’s I was used to test spatial correlation to investigate the spatial effect of air quality governance, the formula is as follows:

(8) $$Moran^{\prime }s\;I=\frac{\sum_{i=1}^{n}\sum_{j=1}^{n}W_{ij}(X_{i}-\bar{X})(X_{j}-\bar{X})}{\sum_{i=1}^{n}\sum_{j=1}^{n}W_{ij}\sum_{i=1}^{n}{\left(X_{i}-\bar{X}\right)}^{2}}=\frac{\sum_{i=1}^{n}\sum_{j=1}^{n}w_{ij}(X_{i}-\bar{X})(X_{j}-\bar{X})}{s^{2}\sum_{i=1}^{n}\sum_{j=1}^{n}W_{ij}}$$

where $s^{2}$ is the variance of X, $\bar{X}$ is the square root of X, *n* is the total number of space units, $W_{ij}$ is the element of the space weight matrix, and $X_{i}$ is the observed value of space unit i.

#### 2.4.3. Variable Selection and Data Sources

##### Air Pollution Governance and Labor Productivity

The explained variable, labor productivity, was measured as the deflated GDP divided by the number of workers (the deflated GDP was based on 2005 data). Air pollution governance is a composite indicator based on various emissions [27]. First, we calculated the emission intensity of air pollution in each city. $I_{ijt}=\frac{P_{ijt}}{Y_{it}}/\frac{1}{n}\sum_{1}^{n}\frac{P_{ijt}}{Y_{it}}$, $I_{ijt}$ was the emission intensity of the type j pollutant in city i and period t. $P_{ijt}$ was the emission of the type j pollutant in city i and period t. $Y_{it}$ was the total industrial output of the city i in period t. Second, the intensity of air pollution emissions was averaged: $I_{it}=\frac{1}{m}\sum_{j=1}^{m}I_{ijt}$. In this study, urban industrial emissions of sulfur dioxide, industrial dust, and PM2.5 were used to measure urban air pollution. Finally, the comprehensive index of air pollution governance was calculated by three kinds of air pollution emissions, $AQG_{it}=\frac{1}{I_{it}}$. The logarithm of air pollution governance was taken to alleviate heteroscedasticity ($\ln AQG_{it}$). The higher the value of $\ln AQG_{it}$, the greater the government’s efforts to govern air pollution.

##### Mediated Variable

The mediated variable in this study was a city’s innovation ability (lnIQ). The number of patents authorized in the Innovation and Entrepreneurship Index of Lungrun Longxin was used to measure a city’s innovation ability. The index is based on more than 50 million records contained in the database of registered industrial and commercial enterprises, and patent and trademark databases. Logarithmic processing was performed to alleviate heteroscedasticity. Another mediating variable was residents’ health (PH). In the existing literature, measurements of residents’ health mostly use neonatal mortality, maternal mortality, and life expectancy. However, there are no statistical data at the city level, so residents’ health is measured by the added value of total medical expenses of urban patients as a proportion of the GDP. The higher the proportion, the lower the health level of urban residents.

##### Control Variables

In this study, the advanced degree of industrial structure (TS) is the output value of the tertiary industry divided by the output value of the secondary industry. Opening to the outside world (FTD) refers to the total import and export trade divided by the GDP. The ratio of expenditures on science and technology (TF) is the proportion of expenditures for science and technology in public finance expenditures. Internet penetration (BB) refers to the number of broadband households divided by the total population. These control variables were used to mitigate the bias caused by omitted variables. The descriptive statistics of specific data are shown in Table 1. Regional differences in labor productivity were evident; the maximum value of 2.096 and the minimum value of 0.015 indicated an imbalance in regional development. China’s air pollution governance gap is relatively large, with a minimum value of 0.182 and a maximum value of 8.175 (average value = 5.631).

In this study, we measured PM2.5 using raster data from the global annual mean of satellite concentrations published by the Center for Socioeconomic Data and Applications at Columbia University [28]. Other data mainly came from the China Statistical Yearbook, China Urban Statistical Yearbook, China Health Statistical Yearbook, official websites of provincial and municipal governments, the Guoyan and EPS websites, and the Peking University Enterprise Big Data Research Center. Missing values were replaced by mean values.

## 3. Results

### 3.1. Dynamic Analysis of Enterprise Productivity

**Figure 6:**
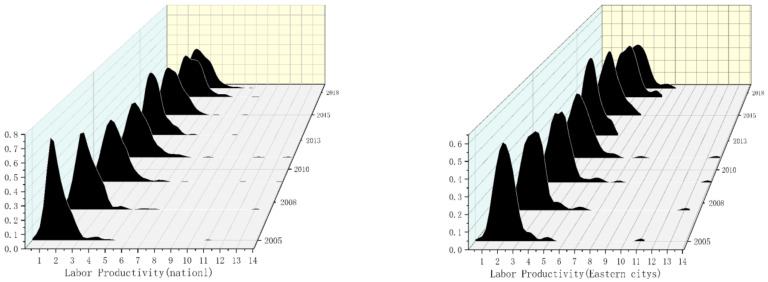
Kernel density of labor productivity in national and eastern cities from 2005 to 2018. Note: the closer the kernel density graph is to the right, the greater the productivity. The higher the kernel density graph, the more concentrated the productivity.

**Figure 7:**
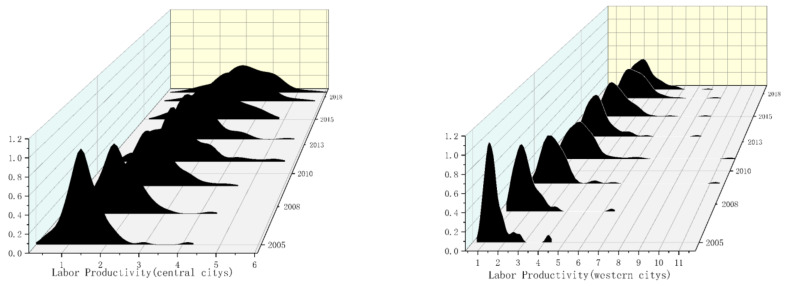
Kernel density of enterprise productivity in central and western cities from 2005 to 2018. Note: the closer the kernel density graph is to the right, the greater the productivity. The higher the kernel density graph, the more concentrated the productivity.

The Gaussian Kernel density function was analyzed to further characterize the dynamic evolution trend of urban labor productivity in China and its eastern, central, and western cities. As shown in Formula (9), f(x) is the density function of labor productivity,

(9) $$f(x)=\frac{1}{Nh}\sum_{i=1}^{N}K(\frac{X_{i}-x}{h})$$

where K(⋅) is the *h*-dimensional kernel, K(⋅) is the product of one-dimensional kernels, N is the number of observed values, X is the mean of the observed value, and h is the optimal bandwidth. The smaller the bandwidth, the higher the estimation accuracy. Figure 6 and Figure 7 respectively show the dynamic changes in labor productivity from 2005 to 2018.

As can be seen from Figure 6, the main peak of the distribution of labor productivity from 2005 to 2018 shifts to the right, indicating that the labor productivity level of all cities was on the rise. Only one main peak is seen for each year, with no flattening trend in the main peak, which indicates the absence of a polarization trend in national labor productivity. Furthermore, regional differences have been shrinking as a whole. As can be seen from Figure 6, the main peak of the distribution of labor productivity in eastern cities is generally broad, indicating that the overall level of labor productivity in eastern cities is relatively balanced. In terms of the value of labor productivity, in eastern cities it is slightly higher than the national average, and the main peak of the eastern cities shifts to the right, indicating that the labor productivity level has improved. The distribution map of labor productivity in eastern cities has only one main peak, and the single peak does not have a flattening trend, which indicates that there is no two-level differentiation phenomenon of labor productivity.

Figure 7 shows that in the distribution of labor productivity in central cities, the main peak shifts to the right, indicating that labor productivity in central cities is significantly improved. There is only one main peak in the distribution map of labor productivity, and it does not have a flattening trend, indicating that there is no two-level differentiation phenomenon of labor productivity in central cities. Further, the regional difference decreases. Figure 7 shows the distribution of labor productivity in western cities, and labor productivity corresponding to the main peak is lower than that of the national, eastern, and central cities, indicating relatively low labor productivity in western cities, but the wave peak gradually shifts to the right, indicating that the labor productivity of western cities increases annually. The annual labor productivity distribution map has only one main peak, and there is no flattening trend in the main peak, indicating that there is no polarization phenomenon. Moreover, the main peak gradually broadens, indicating an imbalance in the distribution of labor productivity in western cities.

### 3.2. Spatial Correlation Analysis of Air Pollution Governance and Labor Productivity

This section mainly discusses the use of Moran’s I to test the spatial correlation between air pollution governance and labor productivity. The specific measurement results are shown in Table 2. All coefficients of air pollution governance and labor productivity passed the significance test, indicating that air pollution governance and labor productivity had a significant spatial effect.

**Figure 8:**
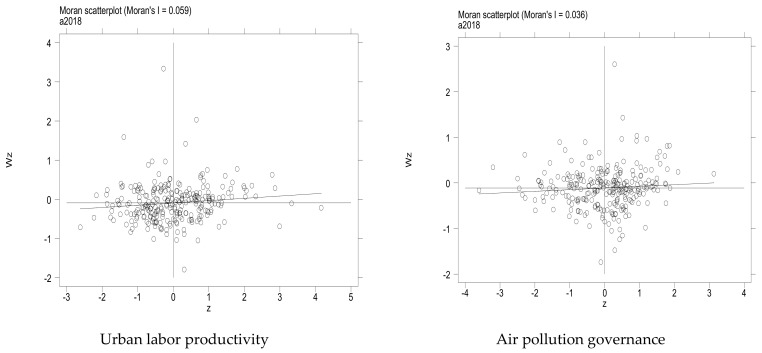
Scatter plot of Moran’s index.

The Moran’s I scatter plot was drawn to observe the spatial accumulation characteristics of air pollution governance and labor productivity. In Figure 8, Moran’s I of air pollution governance and labor productivity is distributed in each quadrant, indicating that the spatial correlation between air pollution governance and labor productivity is strong. The spatial correlation between the two in most cities is represented as high–high, low–low, high–low, and low–high. Significant spatial dependence and high spatial agglomeration characteristics of air quality improvement and labor productivity are indicated.

### 3.3. Analysis of Model Results

The test results from the SDM for air pollution governance are shown in Table 3. The spatial coefficients of the model, ρ or $\beta_{2}$, are significant, and the spatial coefficient of air pollution governance is also significant.

Since ρ or $\beta_{2}$ are not equal to zero in the model, coefficients in the model cannot directly explain the economic significance of variables. Therefore, it is necessary to decompose the effect of air quality improvement (i.e., direct, indirect, and total effects). Decomposition is shown in Table 4.

#### 3.3.1. Direct Effect

Table 4 shows the coefficient of the direct effect is 0.12 and significant at the level of 1%, indicating that air pollution governance can effectively improve regional labor productivity. The reasons are twofold. First, local governments strengthen the control of air pollutant emissions, forcing enterprises to optimize and upgrade production equipment, strengthen their innovation ability, and thus improve labor productivity. Second, the reduction in air pollution emissions can ensure the health level of residents, which can reduce a city’s direct economic losses and avoid the loss of human capital caused by air pollution.

#### 3.3.2. Indirect Effect

Table 4 shows that the indirect effect of air pollution governance—namely, the spatial spillover effect—is significantly positive at the 5% level, which indicates that air pollution governance in neighboring areas can significantly promote the improvement of labor productivity in the region. The theoretical analysis showed that, on the one hand, air pollution governance in neighboring areas forces enterprises to actively develop green production technology and introduce advanced management modes. Because of the spillover of advanced technology, talent and management experience in the neighboring areas will spill over to the region through the cross-regional flow of labor and machinery, leading to improved labor productivity in the region. On the other hand, when air pollution treatment in adjacent areas forces some polluting enterprises to move, the spillover effect of air pollution intensifies, adversely affecting residents’ health, enterprises’ production, and transportation in the region. Therefore, the spillover effect of air pollution governance on labor productivity ultimately depends on the size of the two effects. Table 4 shows that the indirect effect coefficient of air pollution governance is 0.02, which indicates that air pollution governance in adjacent areas promotes labor productivity.

#### 3.3.3. Total Effect

The total effect is the sum of the direct and indirect effects. According to the regression results from the model, the total effect was 0.14 and significant at 1%, indicating that air pollution governance promotes labor productivity. On the one hand, it reduces the health expenditures of residents and the incidence of respiratory diseases, so that the healthy human capital of enterprises is assured. On the other hand, air pollution governance forces local enterprises to upgrade industrial equipment and strengthen their innovation capacity, which improves their competitiveness and labor productivity. Column (3) of Table 3 shows that compared with an ordinary least squares (OLS) estimation without the spatial spillover effect, the SDM effect coefficient of 0.14 is larger than the OLS estimation coefficient of 0.126. This finding also suggests that the OLS estimation underestimates the impact of air pollution governance on labor productivity because it does not consider the spatial spillover effect.

### 3.4. Heterogeneity Analysis

#### 3.4.1. Regional Heterogeneity

The sample was divided into the eastern, central, and western regions to explore the impact of air pollution governance on labor productivity in different regions of China. The results are shown in Table 5.

Table 5 shows that cities in the eastern, central, and western regions all passed the significance test, indicating that air pollution governance in these regions has a significant promoting effect on the improvement of labor productivity. As can be seen from Table 6, from the perspective of the total utility coefficient, the impact coefficient of air pollution governance on labor productivity in the eastern region was 0.160; in the western region it was 0.117, and it was the smallest (0.1) in the central region. The possible reasons are as follows. In the east, where the economy is stronger, the government’s air pollution policy will force companies to upgrade their production facilities. Presently, enterprises are recruiting a large number of talented individuals with scientific and technologically innovative abilities to promote labor productivity. In terms of air pollution governance in the eastern region, some polluting enterprises will move to the central region, which will cause pollution to spread. Therefore, the spatial spillover effect in the central region further aggravates the level of air pollution and significantly hinders improvement in regional labor productivity. As a result, the overall effect of air pollution governance in the central region is relatively low. Because of the low degree of industrial structure in the western region, enterprises use more fossil fuel energy and have lower levels of environmental awareness, causing serious air pollution in the western region. Therefore, when the government implements air pollution governance measures, the promotional effect on labor productivity is more obvious.

#### 3.4.2. Time Heterogeneity

In 2010, China compiled and published technical guidelines on environmental protection standards, technical guidelines for the formulation of standards, and revisions regarding environmental monitoring. These environmental regulations mark China’s more detailed and stringent approach to air pollution governance. Therefore, we took 2010 as the time segmentation point to investigate whether the impact of air pollution governance on labor productivity was different. The results are shown in Table 7.

Table 8 shows that the promoting effect of air pollution governance on labor productivity for the period 2011 to 2018 was significantly greater than that for the period 2005 to 2010, indicating that the promoting effect is more obvious after an increase intensity of air pollution governance.

#### 3.4.3. Heterogeneity of Urban Development Levels

Taking the median per capita GDP of cities as the cutoff point, the sample cities were divided into developed and underdeveloped cities to explore whether air pollution governance had different effects on labor productivity. Table 9 shows that air pollution governance had a significant impact on labor productivity in both developed and underdeveloped cities. The specific effects are shown in Table 10.

From the total effect coefficient, the coefficient of developed cities, 0.137, was significantly higher than that of undeveloped cities (0.101). Developed cities have a better economic foundation. When the government implements air pollution governance, it forces enterprises to introduce green production and emissions technologies, which reduce the resource mismatch rate and promote labor productivity. Underdeveloped areas have a poor economic foundation and a serious loss of innovative talent. When the government implements air pollution governance policies in these areas, enterprises have a limited ability to introduce advanced equipment and innovative talent, which leads to the effect being not as obvious as that in developed cities.

### 3.5. Endogeneity Test

Use of the space instrumental variable method makes it difficult to solve the model’s endogeneity problem because of its complexity and weight matrix endogeneity. Therefore, in addition to controlling for some urban characteristic variables, the instrumental variable method was used to alleviate the endogeneity problems existing in the model. Considering that air pollution governance has significant spatial spillover effects and that air has the nature of diffusion, referring to the research of Broner and Hering [29,30], the air circulation coefficient was chosen as the first instrumental variable of air quality improvement. Environmental regulations do not directly affect enterprise productivity, but the intensity of such regulations will directly affect improvements in the degree of air quality. Therefore, environmental regulation was chosen as the second instrumental variable [31]. For the measurement of environmental regulations, the existing literature mostly uses environmental input, sewage cost, and environmental protection employees [32,33]. It is difficult to reflect on the idea of governance, and to a certain extent, the selection of these indicators is inherent in improving labor productivity. Further, the externality of instrumental variables is difficult to satisfy. Therefore, the frequency of environmental protection words in municipal government reports was used to measure environmental regulations [34,35]. The correlation between environmental regulations and air pollution governance needs no elaboration. Environmental regulations meet externality mainly because government reports are generally released at the beginning of the year, while enterprises’ production activities are carried out throughout the year. Endogeneity problems caused by “reverse causality” can be effectively avoided. Equation (10) shows how to calculate the air circulation coefficient:

(10) VC=WS×BLH

where VC is the air circulation coefficient, WS is the wind speed, and BLH is the atmospheric boundary height. The air flow coefficient is mainly affected by geographical location, such as atmospheric height and the meteorological system. The externality premise of the instrumental variables is well satisfied; the results are shown in Table 11.

In Table 11, the regression results of the first stage show that the two instrumental variables are highly correlated with air pollution governance. The instrumental variables have passed the under-identification, weak identification, and over-identification tests, indicating that the two instrumental variables are valid. According to the results, the coefficient of air pollution governance in the instrumental variable method was 0.830, higher than the regression coefficient of 0.140 in the SDM. Owing to the endogeneity of that model, the impact of air pollution governance on labor productivity is underestimated.

### 3.6. Robustness Tests

Robustness tests were carried out by replacing core explanatory variables, changing spatial weight matrices, and using different spatial models.

#### 3.6.1. Replacement of Core Explanatory Variables

The intensity of three major pollution emissions was divided by the GDP to measure air pollution governance, which expresses a measure of GDP from major pollution emissions. We replaced GDP with gross industrial output, which measures the impact of major polluting emissions on GDP (lnAQG2). Table 12 shows that there is no significant change in the regression results, confirming that the research results are robust and reliable.

#### 3.6.2. Replacement of the Weight Matrix

The study mainly uses a spatial adjacent weight matrix for regression. Here, we used the economic distance weight matrix for the robustness test. The results are shown in Table 13. It can be seen that the coefficient direction and significance of direct, indirect, and total effects do not change. Therefore, it can be concluded that the research conclusion has a certain robustness.

#### 3.6.3. Alternate Regression Method

Since the improvement of labor productivity has a certain time lag, the spatial lag periods L.WLP and L.LP of labor productivity were added to the model as explanatory variables. The dynamic spatial Durbin model (DSDM) was used to investigate the dynamic impact of air pollution governance on labor productivity. Table 14 shows that air pollution governance had a significant spatial effect on the improvement in labor productivity, and both the short-term and long-term effects of air pollution governance promoted such improvement. The research conclusion is consistent with the above results, indicating that it is robust and reliable.

### 3.7. Further Analysis

A mediating effect model was constructed from the two aspects of city innovation capacity and residents’ health to explore the transmission mechanism of air pollution governance in promoting labor productivity.

#### 3.7.1. City Innovation Capacity

To verify that air pollution governance has an impact on labor productivity by influencing urban innovation capacity, we conducted a mediating effect test on labor productivity. Based on Formula (6), Formulas (11) and (12) were developed to express the mediating effect expression of city innovation capability:

(11) $$\begin{array}{l} \ln IQ_{it}=\phi_{0}+\delta W\cdot \ln IQ_{it}+\phi_{1}\ln AQG_{it}+\phi_{2}W\cdot \ln AQG_{it}+\phi_{3}W\cdot X_{control}+\phi_{4}X_{control}\\ +\mu_{i}+\nu_{t}+\varepsilon_{it} \end{array}$$

(12) $$\begin{array}{l} LP_{it}=\alpha_{0}+\delta W\cdot LP_{it}+\alpha_{1}\ln AQG_{it}+\alpha_{2}W\cdot \ln AQG_{it}+\alpha_{3}\ln IQ_{it}+\alpha_{4}W\cdot \ln IQ_{it}\\ +\alpha_{5}W\cdot X_{control}+\alpha_{6}X_{control}+\mu_{i}+\nu_{t}+\varepsilon_{it} \end{array}$$

The regression results are shown in Table 15. Column (1) shows the regression results of the benchmark model Formula (6), and Column (2) shows the regression results of Formula (11). The coefficient of air pollution governance on urban innovation was significant at 0.177, which indicates that air pollution governance can significantly promote an improvement in urban innovation ability. Column (3) provides the regression results of Formula (12). Air pollution governance and urban innovation capacity were entered into the model to investigate whether the mediating effect of city innovation capacity existed. Column (3) shows that the total effect of city innovation capacity on labor productivity is significant at 0.032 (1%), and the air pollution governance coefficient of 0.132 is smaller than the coefficient in Column (1) (0.140) in the benchmark model, indicating the existence of a mediating effect. That is, air pollution governance promotes labor productivity by promoting an improvement in a city’s innovation capacity. In addition, the coefficient of air pollution governance in Column (3) is significantly positive, which indicates that the mediation effect type is incomplete, it means that air pollution governance not only promotes labor productivity by itself but also promotes labor productivity through a city’s innovation ability.

#### 3.7.2. Residents’ Health

The mediating effect model was used to test the effect of air pollution governance on labor productivity by protecting urban residents’ health. Based on Formula (6), Formulas (13) and (14) represent the mediating effect of expressions of residents’ health.

(13) $$\begin{array}{l} PH_{it}=\varphi_{0}+\varphi W\cdot PH_{it}+\varphi_{1}\ln AQG_{it}+\varphi_{2}W\cdot \ln AQG_{it}+\varphi_{3}W\cdot X_{control}+\varphi_{4}X_{control}\\ +\mu_{i}+\nu_{t}+\varepsilon_{it} \end{array}$$

(14) $$\begin{array}{l} LP_{it}=\gamma_{0}+\delta W\cdot LP_{it}+\gamma_{1}\ln AQG_{it}+\gamma_{2}W\cdot \ln AQG_{it}+\gamma_{3}PH_{it}+\gamma_{4}W\cdot PH_{it}+\\ \gamma_{5}W\cdot X_{control}+\gamma_{6}X_{control}+\mu_{i}+\nu_{t}+\varepsilon_{it} \end{array}$$

Specific test results are shown in Table 15; Column (4) shows the regression result of Formula (13). The effect of air pollution treatment on residents’ medical expenses is significantly negative (−0.044) at the 1% level, which indicates that air pollution governance significantly reduces residents’ medical expenses and promotes better health levels. Column (5) shows the regression result of Formula (14). Both air pollution governance and residents’ health were entered into the model to investigate whether the mediating effect of residents’ health existed. Column (5) shows that the influence coefficient of residents’ medical expenses on labor productivity is significant (−0.191) at the 10% level, indicating that the improvement in residents’ health levels significantly promotes an increase in urban labor productivity. Furthermore, the air pollution governance coefficient (0.130) is less than the coefficient of 0.140 in the benchmark model, suggesting the existence of a mediating effect. In other words, air pollution governance promotes the increase in labor productivity by improving the health of residents. In addition, the air pollution governance coefficient in Column (5) is significantly positive, which indicates that the mediating effect type is incomplete. The results show that air pollution governance not only promotes urban labor productivity by itself, but also promotes urban labor productivity by improving residents’ health.

## 4. Discussion

### 4.1. Contribution

The contributions of this study are mainly reflected in the following aspects. First, the existing literature on the correlation between air pollution governance and labor productivity mostly ignores the spatial spillover effect. In this study, the spatial Durbin model was used to investigate the spatial spillover effect of air pollution governance, which was found to promote urban labor productivity by improving urban innovation ability and residents’ health. Second, most existing literature reflect a static perspective. In this study, use of the dynamic spatial Durbin model enabled a robust test from long- and short-term dynamic perspectives. Third, existing literature mostly ignores endogeneity in models when studying air pollution. In this study, the air flow coefficient and environmental regulations were used as instrumental variables to alleviate the bias of research results caused by endogeneity.

### 4.2. Limitations

There are still some limitations in this study, which need to be further investigated. From the aspect of research content, other influence mechanisms should be explored. From the aspect of research methods, this study only examined the spatial effect between air pollution governance and labor productivity, and future research can explore other relationships between them.

## 5. Conclusions

### 5.1. Conclusions

The main conclusion of this study is that air pollution governance significantly promotes an improvement in labor productivity and it also significantly promotes an improvement in regional labor productivity. A city’s innovation capacity and residents’ health are two important mediums for air pollution governance to promote labor productivity increase. In this study, the effect of air pollution governance on labor productivity improvement in eastern cities is better than that in central and western cities, and its effect on labor productivity improvement in developed cities was better than that in undeveloped cities. When the intensity of air pollution treatments increased, the promoting effect on urban labor productivity was more obvious.

### 5.2. Policy Recommendation

The conclusions have a certain guiding significance for effectively implementing air pollution governance measures to promote labor productivity and the construction of an ecological civilization in China. Therefore, the following policy recommendations are presented:

(1) All provinces and cities should form a joint prevention and control mechanism for air pollution governance when implementing government measures due to the obvious spatial spillover effect. In essence, the core of regional joint prevention and governance is that local governments can balance the interests of all regions when forming joint prevention and governmental mechanisms. Therefore, it is necessary for each government to establish a regional emissions trading market and compensation mechanisms, as well as an air pollution emissions detection system to strengthen mutual supervision among regions and promote the formation of joint prevention and control management systems in all provinces and cities. It is also necessary to accelerate the improvement of air quality supervision systems, so that environmental quality can be actually included in the official performance appraisal. Environmental policies must be formulated according to the enterprises’ characteristics, and the implementation of policies must be effective and targeted. Then, environmental policies will ultimately promote an increase in cities’ labor productivity.
(2) The government should implement improved regulations for the prevention and control of total fossil fuel energy consumption. On the one hand, air pollution emissions are controlled from the source. The government has further strengthened air pollution regulation measures to improve air pollution prevention and governance regulations. Further, the government will implement market-oriented policies, such as carbon taxes to enhance the level of intensity in air pollution governance. On the other hand, the government actively encourages enterprises to use clean energy and limits total fossil fuel energy consumption. Through fiscal and tax policies, enterprises are encouraged to actively introduce foreign advanced technologies, innovative talent, and the market competition mechanism is used to force efficient production technologies to gradually replace traditional and backward high-pollution technologies.
(3) The government should implement workers’ security regulations to ensure workers’ welfare and a healthy working environment. On the other hand, a good working environment has gradually become an important non-monetary welfare considered by the labor force. In order to prevent the loss of labor force, enterprises should take the negative effect of air pollution into consideration when formulating management policies. For labor in poor working conditions, enterprises should appropriately increase laborers’ compensation as an alternative compensation, so as to motivate them to improve productivity. Moreover, air pollution will cause productivity losses by harming the health of workers. Therefore, enterprises can increase health insurance for labor to reduce the loss of human capital.
(4) The government makes overall plans based on the levels of air pollution and economic development, and it implements different prevention and control measures. Since the promoting effect of air pollution governance on labor productivity is characterized by significant regional heterogeneity, the focus can be divided into the eastern, central, and western regions. The eastern region is a key economic region in China, and the effect of air pollution governance on labor productivity is obvious, so it can be regarded as a key region for pollution control. Moreover, the strong economic foundation in the eastern region can encourage enterprises to introduce advanced green production and emission technology which can reduce the enterprises’ pollution emission intensity. Since the central region is suffering from the transfer of polluting enterprises from the eastern region, the government can focus on such transfers and improve the relocation standards for enterprises located in the central region. In addition, the central region can establish regional core cities as the transfer stations for the introduction of green technology, and the government can also increase the subsidies for the introduction of green technology to strengthen the spillover effect. To solve the backward economic development and the low degree of urban industrial structure in the western region, the government can provide certain tax subsidies to promote the introduction of green production technology and implement east–west assistance policies to strengthen the spillover effect of green technology and advanced management experience.

## Figures and Tables

**Table 1:** Statistical description of variables.

Variable Name	Mean	Std. Dev.	Median	Min	Max
LP	0.263	0.148	0.241	0.015	2.096
lnAQG	5.631	1.129	5.762	0.182	8.175
TS	0.854	0.398	0.777	0.149	3.458
FTD	0.187	0.327	0.078	0.001	2.882
TF	1.323	1.289	0.927	0.043	11.830
BB	0.152	0.153	0.109	0.006	1.071
VC	7.519	0.526	7.564	4.285	9.102
ER	27.220	14.830	25.000	1.000	108.000
PH	0.059	0.047	0.049	0.001	0.436
lnIQ	6.517	1.706	6.430	2.398	10.920

**Table 2:** Air pollution governance and labor productivity: Moran’s index.

Year	Labor Productivity			Air Pollution Govern		
	I	z	*p*-Value *	I	z	*p*-Value *
2005	0.146	5.133	0.000	0.259	8.34	0.000
2006	0.157	5.577	0.000	0.269	8.655	0.000
2007	0.157	5.562	0.000	0.299	9.596	0.000
2008	0.154	5.480	0.000	0.301	9.653	0.000
2009	0.140	4.864	0.000	0.303	9.714	0.000
2010	0.127	4.413	0.000	0.243	7.837	0.000
2011	0.115	3.970	0.000	0.181	5.882	0.000
2012	0.117	3.995	0.000	0.182	5.893	0.000
2013	0.070	2.370	0.009	0.157	5.111	0.000
2014	0.045	1.574	0.058	0.142	4.632	0.000
2015	0.051	1.757	0.039	0.103	3.397	0.000
2016	0.058	1.984	0.024	−0.048	−1.385	0.083
2017	−0.065	−1.974	0.024	0.047	1.614	0.053
2018	0.059	1.981	0.024	0.036	1.268	0.102

Note: This table reports the Moran’s I estimation results. * denote the significance at 1% levels.

**Table 3:** Spatial Durbin model (SDM) regression estimation results.

Variables	(1)	(2)	(3)
	SDM		OLS
	Main	Wx	
lnAQG	0.120 ***	0.037 ***	0.126 ***
	(0.004)	(0.010)	(0.004)
TS	−0.009	0.059 **	−0.001
	(0.010)	(0.025)	(0.010)
FTD	0.071 ***	0.048	0.066 ***
	(0.014)	(0.032)	(0.014)
TF	−0.011 ***	0.021 ***	−0.011 ***
	(0.002)	(0.005)	(0.002)
BB	−0.099 ***	0.182 ***	−0.082 ***
	(0.022)	(0.058)	(0.023)
rho	−0.128 ***		
	(0.031)		
Time	Yes	Yes	Yes
Region	Yes	Yes	Yes
Obs	3668	3668	3668
Number of city	262	262	262

Note: *** and ** denote the significance at 1%, and 5% levels, respectively.

**Table 4:** Decomposition effect of air pollution governance.

Variable	Direct Effect	Indirect Effect	Total Effect
lnAQG	0.120 ***	0.020 **	0.140 ***
	(0.004)	(0.008)	(0.008)
TS	−0.010	0.054 **	0.044 *
	(0.009)	(0.024)	(0.025)
FTD	0.072 ***	0.035	0.106 ***
	(0.013)	(0.028)	(0.030)
TF	−0.012 ***	0.020 ***	0.009 *
	(0.002)	(0.005)	(0.005)
BB	−0.101 ***	0.176 ***	0.075
	(0.022)	(0.053)	(0.053)

Note: *, **, and *** denote the significance at 10%, 5%, and 1% levels, respectively.

**Table 5:** Impact of air quality improvement on enterprise productivity in eastern, central, and western China.

Variables	(1)	(2)	(3)	(4)	(5)	(6)
	Eastern Cities		Central Cities		Western Cities	
	Main	Wx	Main	Wx	Main	Wx
lnAQG	0.116 ***	0.066 ***	0.124 ***	0.002	0.108 ***	−0.022
	(0.007)	(0.016)	(0.006)	(0.017)	(0.007)	(0.018)
TS	−0.044 **	−0.062	0.065 ***	0.027	−0.035 **	0.116 **
	(0.019)	(0.047)	(0.016)	(0.033)	(0.017)	(0.046)
FTD	0.126 ***	0.069	−0.106 ***	−0.010	−0.010	−0.073
	(0.020)	(0.054)	(0.026)	(0.040)	(0.039)	(0.068)
TF	−0.004	−0.004	−0.003	−0.005	−0.015 ***	−0.007
	(0.004)	(0.009)	(0.003)	(0.008)	(0.005)	(0.008)
BB	−0.074 **	0.123	−0.032	−0.199 *	−0.078 **	0.600 ***
	(0.033)	(0.077)	(0.051)	(0.121)	(0.039)	(0.134)
rho	−0.141 **		−0.269 ***		0.265 ***	
	(0.057)		(0.044)		(0.058)	
Time	Yes	Yes	Yes	Yes	Yes	Yes
Region	Yes	Yes	Yes	Yes	Yes	Yes
Obs	1316	1316	1386	1386	966	966
Number of city	94	94	99	99	69	69

Note: *, **, and *** denote the significance at 10%, 5%, and 1% levels, respectively.

**Table 6:** Decomposition effect of different regions.

Variables	(1)	(2)	(3)	(4)	(5)	(6)	(7)	(8)	(9)
	Eastern Cities			Central Cities			Western Cities		
	Direct Effect	Indirect Effect	Total Effect	Direct Effect	Indirect Effect	Total Effect	Direct Effect	Indirect Effect	Total Effect
lnAQG	0.115 ***	0.045 ***	0.160 ***	0.126 ***	−0.026 **	0.100 ***	0.108 ***	0.009	0.117 ***
	(0.007)	(0.013)	(0.014)	(0.006)	(0.013)	(0.014)	(0.007)	(0.021)	(0.023)
TS	−0.044 **	−0.050	−0.094 *	0.064 ***	0.008	0.072 **	−0.032 *	0.142 **	0.110
	(0.019)	(0.044)	(0.048)	(0.016)	(0.029)	(0.032)	(0.016)	(0.065)	(0.071)
FTD	0.127 ***	0.044	0.171 ***	−0.104 ***	0.014	−0.090 **	−0.009	−0.101	−0.110
	(0.019)	(0.045)	(0.047)	(0.025)	(0.032)	(0.037)	(0.038)	(0.090)	(0.107)
TF	−0.004	−0.003	−0.007	−0.003	−0.003	−0.006	−0.016 ***	−0.014	−0.030 **
	(0.005)	(0.008)	(0.008)	(0.003)	(0.007)	(0.007)	(0.005)	(0.011)	(0.013)
BB	−0.075 **	0.119 *	0.044	−0.023	−0.157	−0.180 *	−0.055	0.774 ***	0.718 ***
	(0.032)	(0.071)	(0.074)	(0.050)	(0.103)	(0.104)	(0.039)	(0.183)	(0.193)

Note: *, **, and *** denote the significance at 10%, 5% and 1% levels, respectively.

**Table 7:** Impact of air quality improvement on enterprise productivity for the periods 2005–2010 and 2011–2018.

Variables	(1)	(2)	(3)	(4)
	2005–2010		2011–2018	
	Main	Wx	Main	Wx
lnAQG	0.042 ***	−0.017	0.150 ***	0.038 ***
	(0.004)	(0.012)	(0.005)	(0.014)
TS	0.011	0.039 **	0.015	0.022
	(0.007)	(0.020)	(0.018)	(0.046)
FTD	−0.025 ***	−0.011	0.077 **	0.091
	(0.008)	(0.019)	(0.032)	(0.065)
TF	−0.007 ***	0.033 ***	−0.008 **	0.005
	(0.002)	(0.003)	(0.004)	(0.009)
BB	−0.006	0.255 ***	0.003	0.161 *
	(0.016)	(0.039)	(0.032)	(0.090)
rho	−0.119 ***		−0.159 ***	
	(0.041)		(0.041)	
Time	Yes	Yes	Yes	Yes
Region	Yes	Yes	Yes	Yes
Obs	1572	1572	2096	2096
Number of city	262	262	262	262

Note: *, **, and *** denote the significance at 10%, 5%, and 1% levels, respectively.

**Table 8:** Decomposition effect of different times.

Variables	(1)	(2)	(3)	(4)	(5)	(6)
	2005–2010			2011–2018		
	Direct Effect	Indirect Effect	Total Effect	Direct Effect	Indirect Effect	Total Effect
lnAQG	0.043 ***	−0.020 *	0.023 **	0.149 ***	0.013	0.163 ***
	(0.004)	(0.010)	(0.011)	(0.005)	(0.011)	(0.011)
TS	0.010 *	0.036 *	0.046 **	0.013	0.018	0.031
	(0.006)	(0.018)	(0.019)	(0.015)	(0.040)	(0.044)
FTD	−0.024 ***	−0.010	−0.034 **	0.078 **	0.060	0.138 **
	(0.008)	(0.016)	(0.017)	(0.034)	(0.051)	(0.057)
TF	−0.007 ***	0.031 ***	0.024 ***	−0.008 **	0.006	−0.003
	(0.002)	(0.003)	(0.003)	(0.004)	(0.008)	(0.009)
BB	−0.013	0.232 ***	0.220 ***	−0.007	0.142 *	0.135
	(0.019)	(0.034)	(0.036)	(0.037)	(0.083)	(0.085)

Note: *, **, and *** denote the significance at 10%, 5%, and 1% levels, respectively.

**Table 9:** Effect of air pollution governance on labor productivity given different urban development levels.

Variables	(1)	(2)	(3)	(4)
	Developed Cities		Underdeveloped Cities	
	Main	Wx	Main	Wx
lnAQG	0.120 ***	0.035 **	0.114 ***	0.001
	(0.006)	(0.014)	(0.005)	(0.014)
TS	−0.038 **	0.058	0.011	0.017
	(0.017)	(0.046)	(0.011)	(0.028)
FTD	0.086 ***	0.070 **	−0.044	−0.057
	(0.017)	(0.035)	(0.029)	(0.054)
TF	−0.008 **	0.023 ***	−0.012 ***	0.004
	(0.003)	(0.007)	(0.004)	(0.009)
BB	−0.131 ***	0.238 ***	0.030	−0.084
	(0.031)	(0.069)	(0.034)	(0.115)
rho	−0.141 ***		−0.150 ***	
	(0.043)		(0.039)	
Time	Yes	Yes	Yes	Yes
Region	Yes	Yes	Yes	Yes
Obs	1820	1820	1848	1848
Number of city	130	130	132	132

Note: ** and *** denote the significance at 5%, and 1% levels, respectively.

**Table 10:** Decomposition effect of different urban development levels.

Variables	(1)	(2)	(3)	(4)	(5)	(6)
	Developed Cities			Underdeveloped Cities		
	Direct Effect	Indirect Effect	Total Effect	Direct Effect	Indirect Effect	Total Effect
lnAQG	0.119 ***	0.017	0.137 ***	0.115 ***	−0.014	0.101 ***
	(0.006)	(0.012)	(0.013)	(0.005)	(0.011)	(0.012)
TS	−0.041 ***	0.059	0.018	0.010	0.014	0.023
	(0.015)	(0.041)	(0.042)	(0.010)	(0.026)	(0.027)
FTD	0.086 ***	0.047 *	0.133 ***	−0.041	−0.052	−0.093 *
	(0.019)	(0.029)	(0.032)	(0.032)	(0.043)	(0.049)
TF	−0.008 **	0.021 ***	0.013 *	−0.012 ***	0.005	−0.007
	(0.003)	(0.007)	(0.007)	(0.004)	(0.008)	(0.009)
BB	−0.141 ***	0.228 ***	0.087	0.025	−0.082	−0.057
	(0.036)	(0.056)	(0.062)	(0.040)	(0.106)	(0.110)

Note: *, **, and *** denote the significance at 10%, 5%, and 1% levels, respectively.

**Table 11:** Instrumental variable regression results.

	(1)	(2)
	First Stage Regression Results	Second Stage Regression Results
Variable	lnAQG	LP
l.lnVL	0.110 *	
	(0.062)	
L.ER	0.009 ***	
	(0.002)	
F value of the first stage regression	22.950	
lnAQG		0.830 ***
		(2.886)
Control variables	Yes	Yes
Observations	3406	3406
Cities	262	262
Under-identification test		*p* = 0.000
Weak identification test		F = 22.952
Over-identification test		*p* = 0.996

Note: This table reports the estimation results of the endogeneity and robustness tests. In column (1), we present the results of the first-stage regression. In column (2), we show the second-stage regression results and effectiveness of instrumental variable selection. Robust standard errors appear in parentheses (). * and *** denote the significance at 10% and 1% levels, respectively.

**Table 12:** Replacement of core explanatory variables.

Variables	(1)	(2)	(3)	(4)	(5)
	Main	Wx	Direct Effect	Indirect Effect	Total Effect
lnAQG2	0.011 **	0.037 ***	0.011 **	0.036 ***	0.046 ***
	(0.005)	(0.012)	(0.005)	(0.011)	(0.012)
TS	−0.029 **	0.126 ***	−0.030 ***	0.123 ***	0.093 ***
	(0.012)	(0.031)	(0.010)	(0.031)	(0.032)
FTD	0.074 ***	−0.011	0.076 ***	−0.020	0.056 *
	(0.016)	(0.036)	(0.017)	(0.031)	(0.034)
TF	−0.009 ***	0.013 **	−0.009 ***	0.013 **	0.004
	(0.003)	(0.006)	(0.003)	(0.006)	(0.006)
BB	−0.117 ***	0.111 *	−0.123 ***	0.111 *	−0.012
	(0.025)	(0.066)	(0.030)	(0.060)	(0.063)
rho	−0.048				
	(0.030)				
Time	Yes	Yes	Yes	Yes	Yes
Region	Yes	Yes	Yes	Yes	Yes
Observations	3668	3668	3668	3668	3668
Number of cities	262	262	262	262	262

Note: *, **, and *** denote the significance at 10%, 5%, and 1% levels, respectively.

**Table 13:** Weight matrix of economic distance.

Vairable	(1)	(2)	(3)	(4)	(5)
	Main	Wx	Direct Effect	Indirect Effect	Total Effect
lnAQG	0.120 ***	0.320 ***	0.119 ***	0.236 ***	0.355 ***
	(0.004)	(0.072)	(0.004)	(0.048)	(0.049)
TS	0.000	0.305 **	−0.000	0.250 *	0.250 *
	(0.010)	(0.154)	(0.009)	(0.141)	(0.142)
FTD	0.063 ***	−0.300	0.065 ***	−0.261	−0.197
	(0.014)	(0.260)	(0.013)	(0.207)	(0.208)
TF	−0.010 ***	0.002	−0.010 ***	0.006	−0.004
	(0.002)	(0.030)	(0.002)	(0.025)	(0.025)
BB	−0.090 ***	−0.046	−0.089 ***	−0.019	−0.108
	(0.022)	(0.439)	(0.022)	(0.368)	(0.367)
rho	−0.250				
	(0.184)				
Time	Yes	Yes	Yes	Yes	Yes
Region	Yes	Yes	Yes	Yes	Yes
Observations	3668	3668	3668	3668	3668
Number of cities	262	262	262	262	262

Note: *, **, and *** denote the significance at 10%, 5%, and 1% levels, respectively.

**Table 14:** Alternate regression method: the dynamic spatial Durbin model (DSDM).

Variables	(1)	(2)	(3)	(4)	(5)	(6)	(7)	(8)
	Main	Wx	Short-Term Direct Effect	Short-Term Indirect Effect	Short-Term Total Effect	Long-Term Direct Effect	Long-Term Indirect Effect	Long-Term Total Effect
L.LP	0.247 ***							
	(0.015)							
L.WLP	0.028							
	(0.036)							
lnAQG	0.125 ***	0.026 **	0.125 ***	0.008	0.133 ***	0.166 ***	0.009	0.175 ***
	(0.004)	(0.010)	(0.004)	(0.008)	(0.008)	(0.005)	(0.011)	(0.011)
TS	−0.001	0.022	−0.001	0.021	0.020	−0.002	0.028	0.026
	(0.011)	(0.027)	(0.010)	(0.024)	(0.025)	(0.014)	(0.031)	(0.033)
FTD	0.056 ***	0.067 **	0.055 ***	0.055 *	0.110 ***	0.073 ***	0.073 *	0.145 ***
	(0.015)	(0.034)	(0.014)	(0.030)	(0.032)	(0.019)	(0.040)	(0.042)
TF	−0.007 ***	0.013 **	−0.007 ***	0.012 **	0.005	−0.009 ***	0.016 **	0.007
	(0.003)	(0.006)	(0.003)	(0.005)	(0.006)	(0.003)	(0.007)	(0.007)
BB	−0.061 ***	0.164 ***	−0.064 ***	0.158 ***	0.094	−0.086 ***	0.210 ***	0.124
	(0.023)	(0.059)	(0.024)	(0.056)	(0.058)	(0.032)	(0.074)	(0.076)
rho	0.135 ***							
	(0.032)							
Time	Yes	Yes	Yes	Yes	Yes	Yes	Yes	Yes
Region	Yes	Yes	Yes	Yes	Yes	Yes	Yes	Yes
Obs	3668	3668	3668	3668	3668	3668	3668	3668
Number of city	262	262	262	262	262	262	262	262

Note: *, **, and *** denote the significance at 10%, 5%, and 1% levels, respectively.

**Table 15:** Air pollution governance and labor productivity: a mediating effect test.

Variables	(1)	(2)	(3)	(4)	(5)
	LP	lnIQ	LP	PH	LP
lnAQG	0.140 ***	0.177 ***	0.132 ***	−0.044 ***	0.130 ***
	(0.008)	(0.035)	(0.007)	(0.004)	(0.008)
lnIQ			0.032 ***		
			(0.010)		
PH					−0.191 *
					(0.104)
TS	0.044 *	−0.296 ***	0.062 ***	−0.009	0.041 *
	(0.025)	(0.106)	(0.023)	(0.010)	(0.023)
FTD	0.106 ***	0.012	0.098 ***	−0.039 ***	0.093 ***
	(0.030)	(0.126)	(0.034)	(0.012)	(0.034)
TF	0.009 *	0.061 ***	0.004	0.001	0.008
	(0.005)	(0.021)	(0.006)	(0.002)	(0.006)
BB	0.075	−1.109 ***	0.092	−0.000	0.076
	(0.053)	(0.222)	(0.059)	(0.020)	(0.058)
W·lnAQG	0.037 ***	0.132 ***	0.031 ***	−0.008 **	0.043 ***
	(0.010)	(0.034)	(0.010)	(0.003)	(0.011)
W·lnIQ			0.030 ***		
			(0.011)		
W·PH					0.326 ***
					(0.121)
rho	−0.128 ***	−0.003	−0.126 ***	0.070 **	−0.110 ***
	(0.031)	(0.030)	(0.031)	(0.031)	(0.031)
Time	Yes	Yes	Yes	Yes	Yes
Region	Yes	Yes	Yes	Yes	Yes
Obs	3668	3668	3668	3668	3668
Number of cities	262	262	262	262	262

Note: To clearly show the effects of air pollution governance and mediating variables on labor productivity, the results reported in this table are the total effects. *, **, and *** denote the significance at 10%, 5%, and 1% levels, respectively.

## Data Availability

The Ministry of Ecology and Environment of the People’s Republic of China (https://www.mee.gov.cn/, accessed on 15 August 2022); the National Bureau of Statistics (http://www.stats.gov.cn/, accessed on 16 August 2022); China National Intellectual Property Administration (https://www.cnipa.gov.cn/, accessed on 16 August 2022); and EPSDATA (https://www.epsnet.com.cn/index.html#/, Home, accessed on 17 August 2022).
